# Supplementary material for: Age-specific biological and molecular profiling distinguishes paediatric from adult acute myeloid leukaemias
Source: Nat Commun. 2018 Dec 11;9:5280. doi: 10.1038/s41467-018-07584-1 (PMC6290074; doi:10.1038/s41467-018-07584-1)
Supplement: Supplementary file 1 — Supplementary Information [file 41467_2018_7584_MOESM1_ESM.pdf]

Title

Age-specific biological and molecular profiling distinguishes paediatric from adult acute myeloid leukaemias

Chaudhury et al

Supplementary Figures

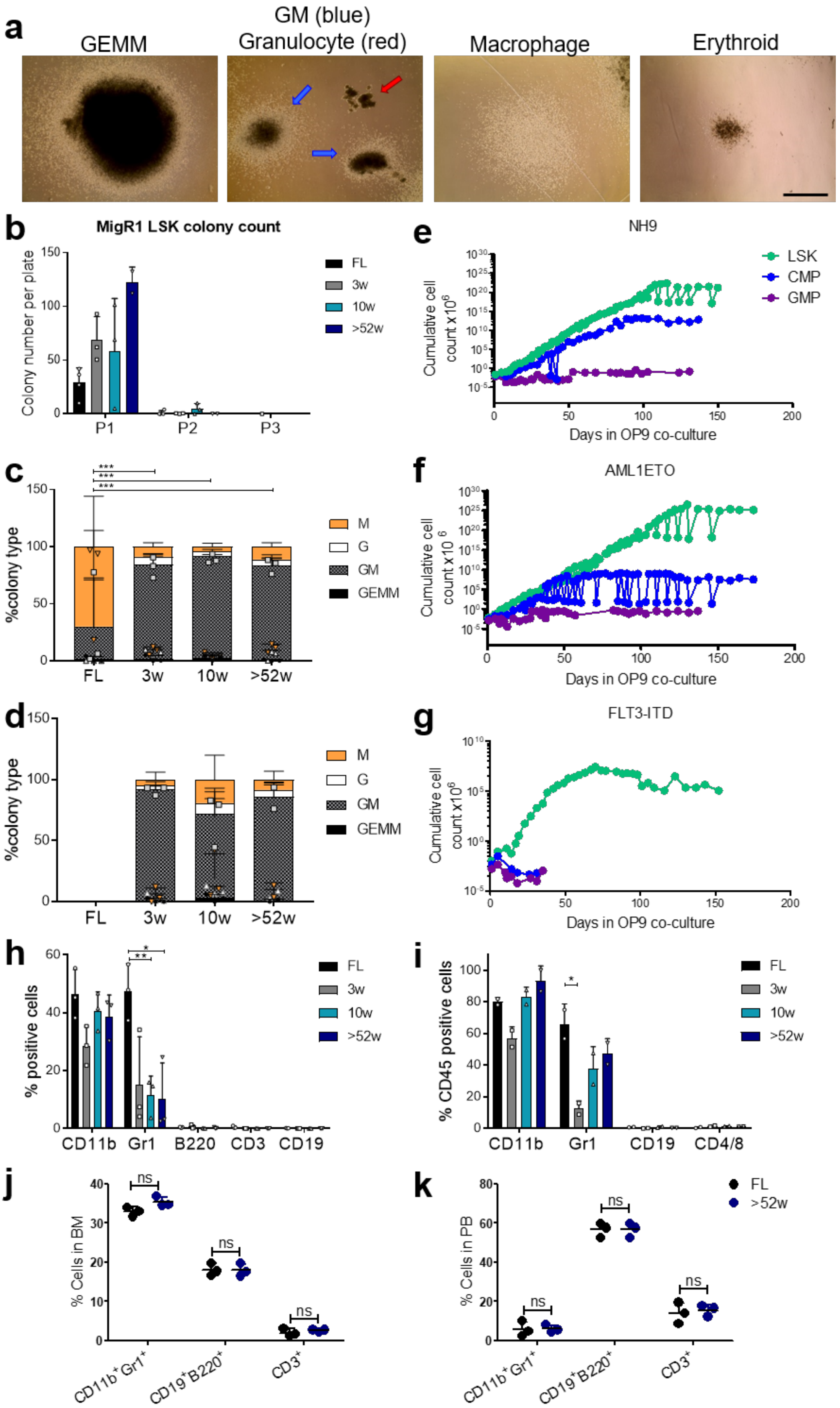

a
GEMM
GM (blue)
Granulocyte (red)
Macrophage
Erythroid
b
MigR1 LSK colony count
Colony number per plate
P1
P2
P3
FL
3w
10w
>52w
c
%colony type
FL
3w
10w
>52w
M
G
GM
GEMM
d
%colony type
FL
3w
10w
>52w
M
G
GM
GEMM
e
NH9
Cumulative cell count x10^6
Days in OP9 co-culture
LSK
CMP
GMP
f
AML1ETO
Cumulative cell count x10^6
Days in OP9 co-culture
g
FLT3-ITD
Cumulative cell count x10^6
Days in OP9 co-culture
h
% positive cells
CD11b
Gr1
B220
CD3
CD19
FL
3w
10w
>52w
i
% CD45 positive cells
CD11b
Gr1
CD19
CD4/8
FL
3w
10w
>52w
j
% Cells in BM
CD11b+Gr1+
CD19+B220+
CD3+
ns
k
% Cells in PB
CD11b+Gr1+
CD19+B220+
CD3+
ns
FL
>52w

Supplementary Figure 1: Analysis of normal and transformed cells of different ages *in vitro* and *in vivo*. (a) Representative photographs of GEMM, GM, granulocyte, macrophage and erythroid colonies (taken at x4 magnification, black scale bar represents 1mm). (b) Graph of number of colonies produced per plate in CFC assay with LSKs transduced with MigR1 control. Bars depict mean +/- SD. FL n=4, 3w n=3, 10w n=3, >52w n=3. Graph of percentage colony type produced at P3 from NH9 transduced (c) CMPs and (d) GMPs. Granulocyte erythroid macrophage megakaryocyte (GEMM), granulocyte macrophage (GM), G=granulocyte and M=macrophage. Graphs depict mean percentage +SD. Significance determined by 2-way ANOVA and Bonferroni post-test, ***p<0.001. FL n=4, 3w n=3, 10w n=3, >52w n=3. Growth of (e) NH9 (n=2), (f) AML1ETO (n=3) and (g) FLT3-ITD (n=2) transduced FL-LSKs, CMPs and GMPs grown on stromal OP9 co-culture. (h) Graph of myeloid and lymphoid surface marker expression in cells at P2 showing no expression of lymphoid markers (FL, 3w, 10w, >52w (n=3)) and (i) after 4 days in co-culture with OP9 cells showing cells from all ages were differentiated into myeloid cells (FL, 3w, 10w, >52w (n=2)). Significance determined by Student's t-test, *p<0.05, **p<0.01. Graphs shown log2 mean cumulative cell number normalised to GFP%, n=2. Graph of the distribution of myeloid ($CD11b^+Gr\text{-}1^+$), B ($B220^+CD19^+$) and T ($CD3^+$) cells in the (j) BM and (k) PB of 6-8wk mice transplanted with healthy LSKs isolated from FL (LSK^{FL}) (n=3) or >52w BM ($LSK^{>52W}$) (n=3). Dots represent individual mice with average +/- SD displayed. ns=not significant as determined by Student's t-test.

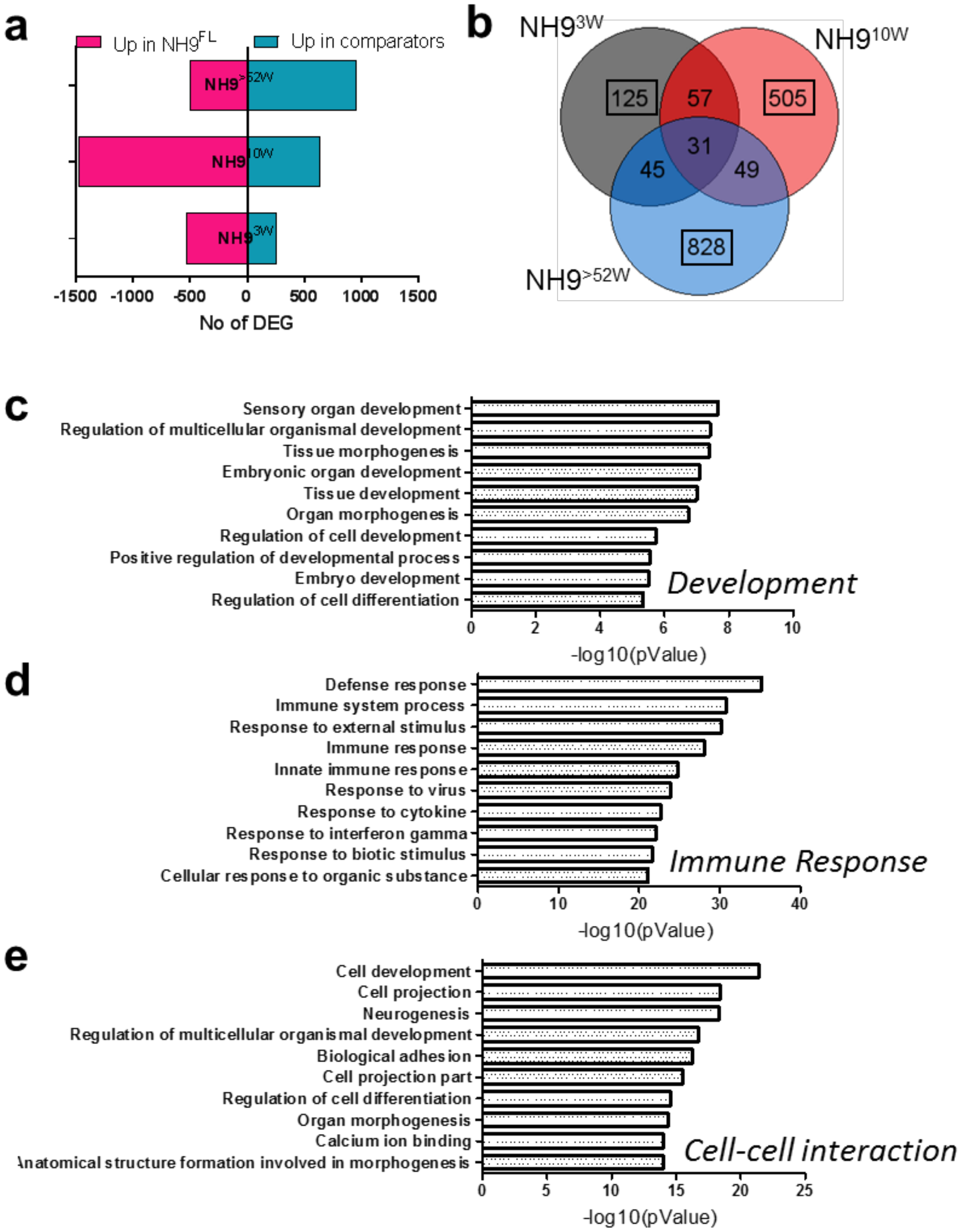

Supplementary Figure 2: Analysis of DEGs in $NH9^{3w}$, $NH9^{10w}$ and $NH9^{>52w}$ compared to $NH9^{FL}$. (a) Summary plot of DEGs in $NH9^{3w}$, $NH9^{10w}$ and $NH9^{>52w}$ compared to $NH9^{FL}$. DEGs are defined as $q<0.05$ and $FC \geq 1.5$. $n=3$ for all groups. (b) Venn diagram of overlapping upregulated DEGs in $NH9^{3w}$, $NH9^{10w}$ and $NH9^{>52w}$ compared to $NH9^{FL}$. Genes highlighted in white boxes were used for pathway analysis (c-e). Top ten significantly enriched GO (Biological Process) pathways using DEGs upregulated solely in (c) $NH9^{3w}$, (d) $NH9^{10w}$ and (e) $NH9^{>52w}$ compared to $NH9^{FL}$.

Analysis was performed on MSigDB using the C5 module, with FDR q-value set to <0.05. Significant pathway enrichment graphed as $–\log^{10}$(p-value).

a

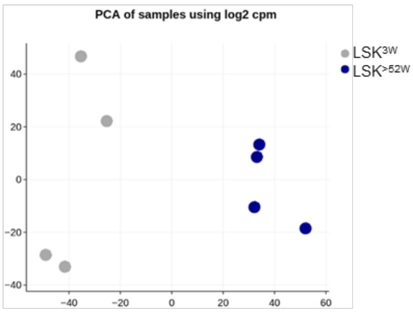

b

Gene Set Name	Enrichment	p-value
Small molecule metabolic process	18/1767	1.32^{e-6}
Response to endogenous stimulus	16/1450	1.97^{e-6}
Regulation of phosphorus metabolic process	16/1618	7.85^{e-6}
Regulation of camp metabolic process	5/129	2.78^{e-5}
Regulation of cyclic nucleotide metabolic process	5/155	6.67^{e-5}
Lipid metabolic process	12/1158	7.41^{e-5}
Positive regulation of camp metabolic process	4/89	1.03^{e-4}
Forebrain neuron development	3/34	1.08^{e-4}
Regulation of transport	15/1804	1.1^{e-4}
Neurogenesis	13/1402	1.12^{e-4}

c

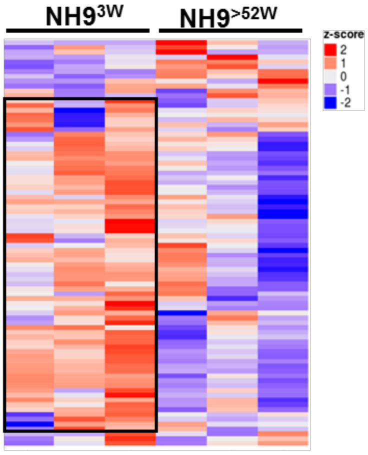

d

Gene Set Name	Enrichment	p-value
Biological adhesion	33/1032	1.22^{e-22}
Tissue development	32/1518	1.06^{e-16}
Extracellular structure organization	17/304	5.43^{e-16}
Immune system process	34/1984	4.55^{e-15}
Organ morphogenesis	23/841	1.59^{e-14}
Cellular response to organic substance	32/1848	2.51^{e-14}
Response to external stimulus	31/1821	1.06^{e-13}
Cell proliferation	20/672	1.99^{e-13}
Regulation of cellular component movement	21/771	2.61^{e-13}
Cell cell adhesion	19/608	3.53^{e-13}

e

Gene Set Name	Enrichment	p-value
Biological adhesion	66/1032	3.92^{e-28}
Tissue development	79/1518	1.18^{e-27}
Regulation of multicellular organismal development	81/1672	2.64^{e-26}
Regulation of cell differentiation	74/1492	1.17^{e-24}
Cell development	71/1426	9.06^{e-24}
Positive regulation of multicellular organismal process	70/1395	1.24^{e-23}
Regulation of transport	80/1804	1.52^{e-23}
Organ morphogenesis	54/841	3.19^{e-23}
Positive regulation of developmental process	61/1142	5.07^{e-22}
Embryo development	54/894	5.37^{e-22}

f

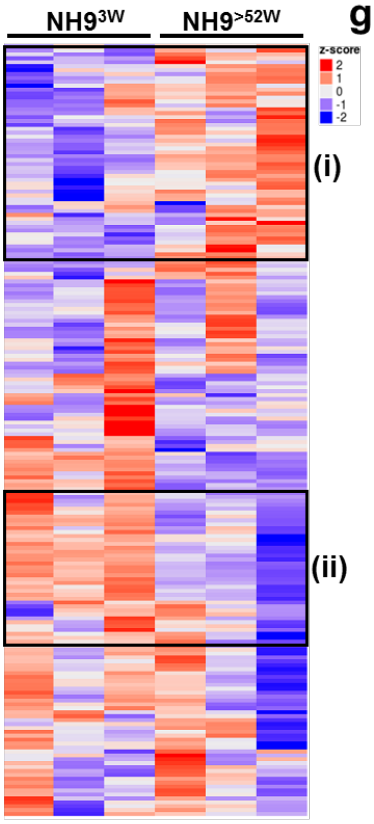

g

Cluster (i)

Il11ra1	Itga2b
Itga9	Igf1r
Notch4	Gp1ba
Crlf2	Il18r1
Igf1	Notch3
Pdgfrb	Acvr2a
Nlgn2	Bmpr2
Egf	F2rl2
Plp1	Icam5
Cd72	Cd36
Cd226	Sele
Fzd6	Eng
Ephb6	Tnfrsf12a
Ccr7	Epha2
Itga2	Il12rb2
Il9r	Lrp6
Efnb3	F2rl3
Jam3	Tnfrsf10b
Nlgn3	Kit
C3ar1	Acvr2b
Acvr1	Flt3
Plxnc1	Erbb3
Epor	Masp2
Tgfbr3	Selp
Tek	Il5ra
F2r	Fzd1
F8	Cr2
Ptch1	

Cluster (ii)

Acvr1b	F10
Tgfbr1	Ifnar2
Itgav	Itga8
Fgfr2	Itga1
Il6st	Il1r1
Cd28	Tnfrsf1b
Lrp5	Il6ra
Tnfrsf13b	Itgb3
Il21r	Sdc2
Nrp2	Cldn1
Ccr5	F5
Ptch2	Efnb2
C3	Hc
Tnfsf13	Itgal
Nrp1	Adipor2
Plxnd1	Tnfrsf8
Ifngr1	Cd44
Ltbr	Plaur
Lifr	Acvrl1
Csf1r	Cxcr4

Supplementary Figure 3: Hierarchical clustering analysis of NH9^{3w} and NH9$^{>52w}$ RNA seq data. (a) Plot of principal component analysis of gene expression in LSK^{3W} and LSK$^{>52W}$ cells. n=4 in each group. (b) Top ten enriched GO pathways using DEGs upregulated in NH9$^{>52W}$ samples compared to NH9^{3W} but not in LSK$^{>52W}$ samples. Analysis performed on MSigDB using C5 module (GO Biological Processes) with an FDR q-value of <0.05. The RNA seq dataset containing gene expression data from (n=3) NH9^{3w} and (n=3) NH9$^{>52w}$ myeloid leukaemia blasts was analysed using the

Stemformatics data analysis platform. (c) Hierarchical clustering analysis of the KEGG Toll-like receptor signalling pathway gene set (100 genes) highlighting enrichment in NH9^{3w} samples. Top ten enriched GO pathways using DEGs upregulated in (d) LSK^{3W} samples compared to LSK$^{>52W}$ but not NH9^{3W} samples and (e) LSK$^{>52W}$ samples compared to LSK^{3W} but not NH9$^{>52W}$ samples. Analysis performed on MSigDB using C5 module (GO Biological Processes) with an FDR q-value of <0.05. (f) Hierarchical clustering analysis of a gene-signature for receptor-associated genes in cancer-stroma interactions identifying 2 enriched clusters (i,ii). Heat maps (a, f) depict colour-coded (blue-red, low-high) TMM RPKM expression values. (g) Gene cluster (i) upregulated in NH9$^{>52w}$ compared to NH9^{3w}, as indicated in (f). Gene cluster (ii) upregulated in NH9^{3w} compared to NH9$^{>52w}$, as indicated in (f).

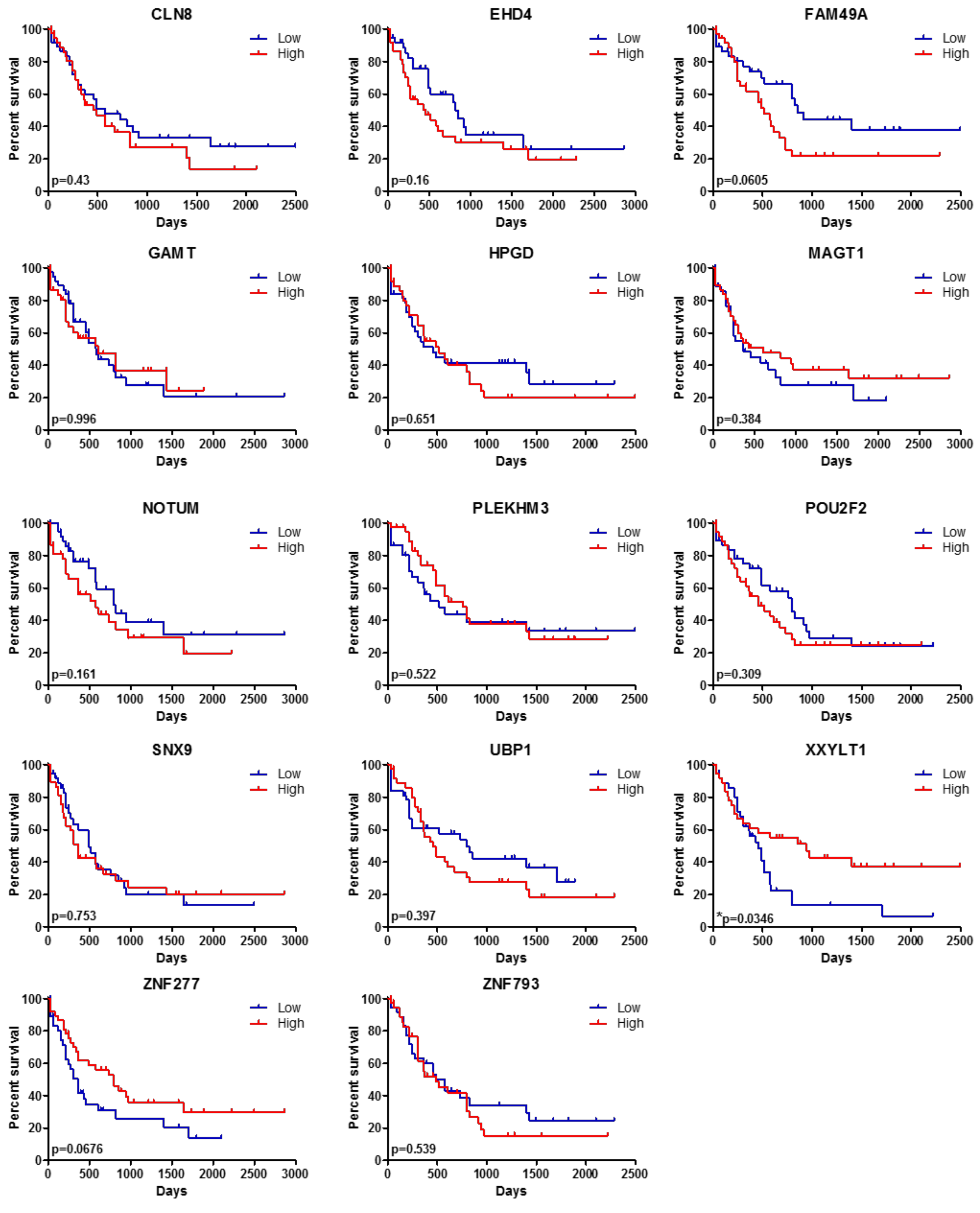

Supplementary Figure 4: Prognostic significance of genes significantly upregulated in human paediatric AML. Kaplan-Meier survival analysis of overall survival of AML patients (n=150) in the TCGA LAML dataset with high (top quartile, n=37) and low (bottom quartile, n=37) expression of genes identified in **Fig 5h**. Significance determined by overall log rank *p<0.05.

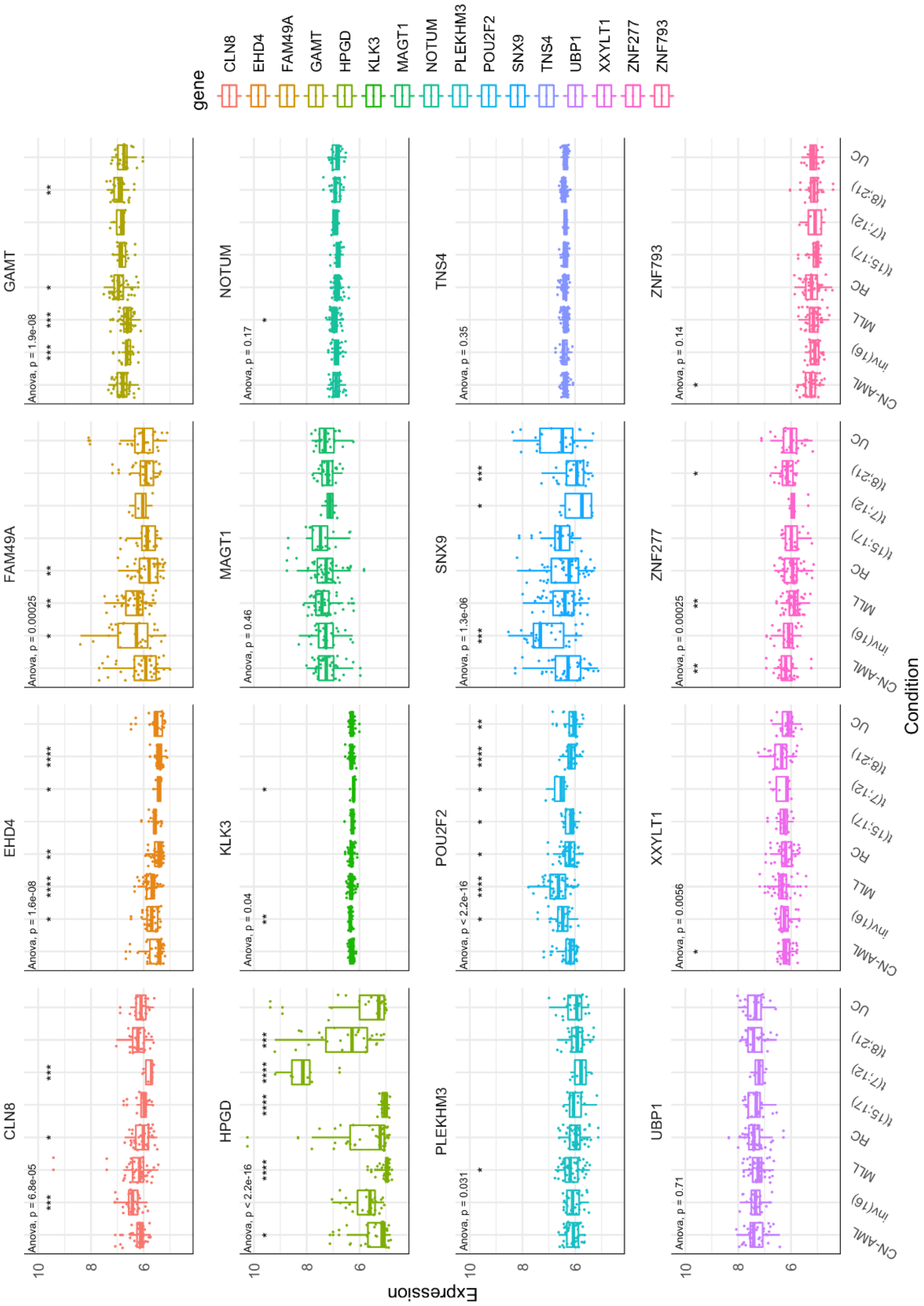

Supplementary Figure 5: Expression of genes significantly upregulated in human paediatric AML categorised by disease subtype. Expression of genes from human paediatric gene signature (**Fig 5h**) in paediatric AML patients (GSE17855) categorised by AML subtypes - cytogenetically normal AML (CN-AML, n=39), AML with inv(16) (n=27), MLL rearranged AML (MLL, n=47), AML with remaining cytogenetics including t(6;9), monosomy 7, trisomy 8 and complex karyotype (RC, n=45), AML with translocations t(15;17) (n=19), t(7;12) (n=7) and t(8;21) (n=28) and

patients with unknown cytogenetics (UC, n=25). Significance determined by ANOVA *p<0.05, **p<0.01, ***p<0.001, ****p<0.0001.

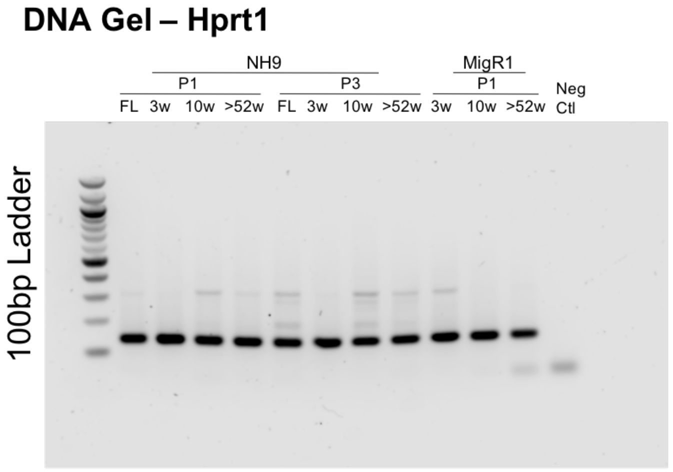

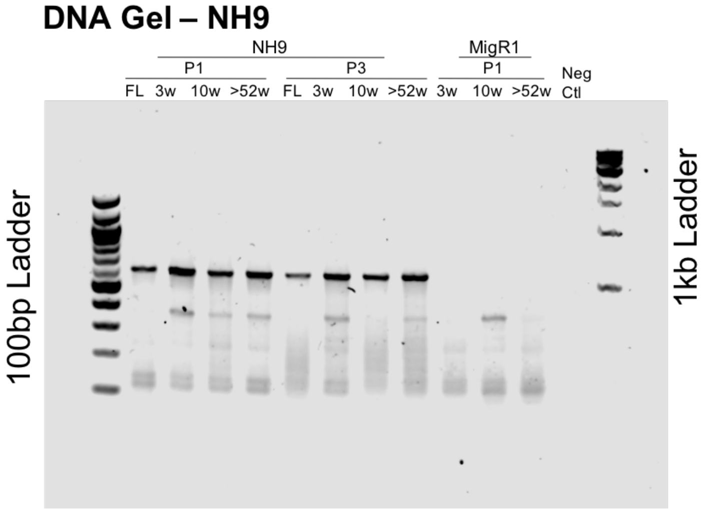

Supplementary Figure 6: Uncropped gels from Figure 1c.

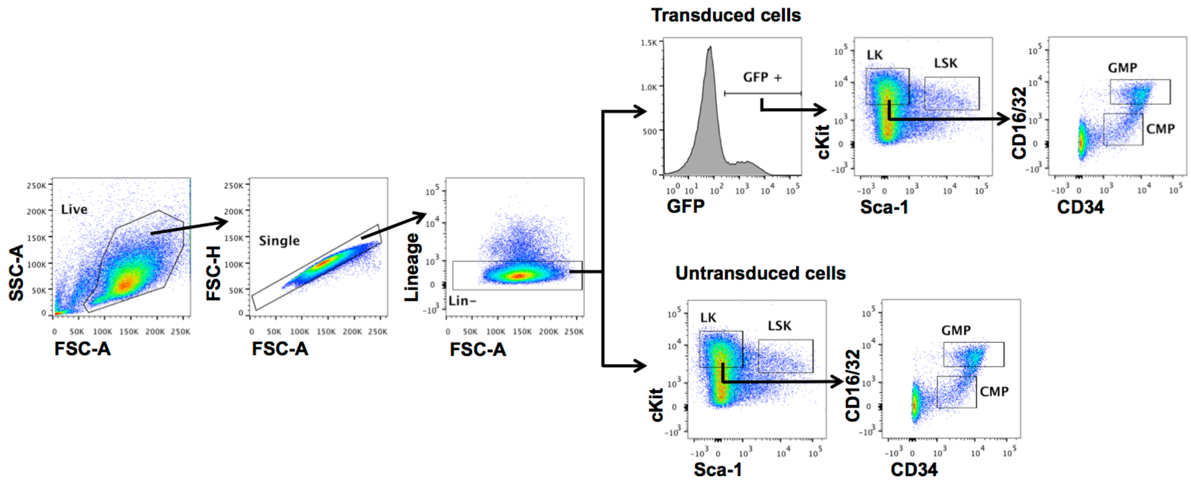

Supplementary Figure 7: Strategy for sorting transduced (top) and untransduced (bottom) LSKs, CMPs and GMPs.

Primers	Sequences
NH9	Forwards: TAATGAATTCCGGGCTCTTTGGTTTTGGCA<br>Reverse: TAA GGT ACC GCA TCA CTC GTC TTT TGC TCG
Hprt1	Forward: GAGAGCGTTGGGCTTACCTC<br>Reverse: ATCGCTAATCACGACGCTGG
Cebpβ	Forward: CCGGATCAAACGTGGCTGAG<br>Reverse: CACGTGTGTTGCGTCAGTC
Cebpε	Forward: GAGGCAGCTACAATCCCCTG<br>Reverse: CACAGGGGCCTTGAGGACA
Fcgr3	Forward: TCTGCTGCTGTTTGCTTTTGC<br>Reverse: CCAGTTTCACCACAGCCTTC
Cd79a	Forward: GTCATACGCCTGTTTGGGTCC<br>Reverse: AAGGCTGAACCACCATGTGA
Ptprc	Forward: GTCACAGGGCAAACACCTACA<br>Reverse: AGGGCATTCTCTGTTGTGCTC
Pou2af1	Forward: CCTCGGTGTTGACCTATGCT<br>Reverse: CAGTGCTTCTTGGCGTGACA
Foxo1	Forward: GAGTTAGTGAGCAGGCTACATTT<br>Reverse: TTGGACTGCTCCTCAGTTCC
Vpreb1	Forward: CTGGACGTCTGTCCTGCTCA<br>Reverse: AGGGCCACAACCTGTGAGATAG
Rag1	Forward: GTTGCTATCTCTGTGGCATCG<br>Reverse: TAAGCTACCTTGCTCCACAGG
Rag2	Forward: AGTGACTCTTCCCCAAGTGC<br>Reverse: TTCTTATTTTGGCACTGAAGGC
Il7rα	Forward: GGCCTAGTCTCCCCGATCA<br>Reverse: TTCAGACTCGTTTTTGGCTTCT
Flt3	Forward: TTGGCCTTTGTGTCTTCCGT<br>Reverse: TTGCGAGCTGGTAGCGTTTA

Supplementary Table 1: List of primers including names and sequences
