# Supplementary material for: Age-specific biological and molecular profiling distinguishes paediatric from adult acute myeloid leukaemias
Source: Nat Commun. 2018 Dec 11;9:5280. doi: 10.1038/s41467-018-07584-1 (PMC6290074; doi:10.1038/s41467-018-07584-1)
Supplement: Supplementary file 2 — Description of Additional Supplementary Files [file 41467_2018_7584_MOESM2_ESM.docx]

Description of Additional Supplementary Files

Supplementary Data 1: List of DEGs upregulated in both NH9^3W^ compared to NH9^>52W^ and LSK^3W^ compared to LSK^>52W^ (3w intersect)

Supplementary Data 2: List of DEGs upregulated in both NH9^>52W^ compared to NH9^3W^ and LSK^>52W^ compared to LSK^3W^ (>52w intersect)

Supplementary Data 3: List of DEGs upregulated in NH9^3W^ compared to NH9^>52W^ minus 3w intersect

Supplementary Data 4: List of DEGs upregulated in NH9^>52W^ compared to NH9^3W^ minus >52w intersect

Supplementary Data 5: List of DEGs upregulated in LSK^3W^ compared to LSK^>52W^ minus 3w intersect

Supplementary Data 6: List of DEGs upregulated in LSK^>52W^ compared to LSK^3W^ minus >52w intersect

Supplementary Data 7: List of genes present in GO pathways identified using DEGs upregulated in NH9^3W^ compared to NH9^>52W^ samples but not in LSK^3W^

Supplementary Data 8: List of genes present in GO pathways identified using DEGs upregulated in NH9^>52W^ samples compared to NH9^3W^ but not in LSK^>52W^ samples

Supplementary Data 9: List of genes present in GO pathways identified using DEGs upregulated in LSK^3W^ samples compared to LSK^>52W^ but not NH9^3W^ samples

Supplementary Data 10: List of genes present in GO pathways identified using DEGs upregulated in LSK^>52W^ samples compared to LSK^3W^ but not NH9^>52W^ samples
